# Understanding the Multiple Role of Mitochondria in Parkinson’s Disease and Related Disorders: Lesson From Genetics and Protein–Interaction Network

**DOI:** 10.3389/fcell.2021.636506

**Published:** 2021-04-01

**Authors:** Valentina Nicoletti, Giovanni Palermo, Eleonora Del Prete, Michelangelo Mancuso, Roberto Ceravolo

**Affiliations:** Unit of Neurology, Department of Clinical and Experimental Medicine, University of Pisa, Pisa, Italy

**Keywords:** Parkinson’s disease, atypical parkinsonism, Huntington disease, mitochondrial dysfunction, neurodegenerative diseases, Pink1/parkin pathway, alpha-synuclein

## Abstract

As neurons are highly energy-demanding cell, increasing evidence suggests that mitochondria play a large role in several age-related neurodegenerative diseases. Synaptic damage and mitochondrial dysfunction have been associated with early events in the pathogenesis of major neurodegenerative diseases, including Parkinson’s disease, atypical parkinsonisms, and Huntington disease. Disruption of mitochondrial structure and dynamic is linked to increased levels of reactive oxygen species production, abnormal intracellular calcium levels, and reduced mitochondrial ATP production. However, recent research has uncovered a much more complex involvement of mitochondria in such disorders than has previously been appreciated, and a remarkable number of genes and proteins that contribute to the neurodegeneration cascade interact with mitochondria or affect mitochondrial function. In this review, we aim to summarize and discuss the deep interconnections between mitochondrial dysfunction and basal ganglia disorders, with an emphasis into the molecular triggers to the disease process. Understanding the regulation of mitochondrial pathways may be beneficial in finding pharmacological or non-pharmacological interventions to delay the onset of neurodegenerative diseases.

## Introduction

Neurodegenerative diseases represent one of the major challenges of our era. Despite their high frequency in populations and impact on our society, pathogenic mechanisms are still widely unknown, and therapeutic approach are only symptomatic nowadays. Movement disorders, together with dementias, are the most frequent neurodegenerative diseases in elderly with Parkinson’s disease (PD) affecting up to 2% of the population over 65 years (Ray Dorsey et al., 2018). It is characterized by the progressive loss of dopaminergic neurons in pars compacta of substantia nigra (SN) and the presence of intracellular aggregates of the protein α-synuclein (α-syn). PD is sporadic in most cases (90–95%) but inherited in approximately 5–10% of cases (Bonifati, 2007; Gasser, 2009; Hernandez et al., 2016). The main clinical features are bradykinesia, rigidity, resting tremor, and gait disturbance, preceded and associated with no-motor symptoms such as rapid eye movement (REM) behavior disorder, hyposmia, and depression.

Although there are many efforts of research in investigating the pathogenesis of PD, primary causes of this disease remain elusive. Mitochondria have often been proposed as potential candidates involved in neurodegeneration. Indeed, the main risk factor for neurodegenerative disease as well as PD is aging in which mitochondria play an important role (Sun et al., 2016; Giannoccaro et al., 2017; Rango and Bresolin, 2018; Theurey and Pizzo, 2018). Experimental models (Schwarze et al., 1995; Khaidakov et al., 2003) and postmortem studies in elderly (Corral-Debrinski et al., 1992; Ojaimi et al., 1999) exhibited an increased load of mitochondrial DNA (mtDNA) mutations and deletions and a reduced respiratory chain activity. The main challenge is establishing whether mitochondria impairment could represent the initial cause of neurodegeneration or an epiphenomenon (Borsche et al., 2020). This review aims to describe the evidence, particularly from genetic field, which highlight the critical role of these organelles in participating and fostering the neurodegenerative processes, especially PD and other neurodegenerative movement disorders. Then, it shows the importance of the relationship between mitochondrial dysfunction and deposits of aggregated α-syn and between mitochondrial dysfunction and impairment of other cellular degradative pathways, particularly lysosomal system.

## PD and Energy Production Function of Mitochondria

For a long time, the pathogenic role of mitochondria in PD has mainly been considered linked to their function of energy producers for cells and the consequent effect of generators of reactive oxygen species (ROS). Indeed in humans, the brain is the most energy-demanding organ, accounting for approximately 20% of the body’s total demand (Clarke and Sokoloff, 1999). Mitochondrial ATP production is very important for multiple neuronal processes such as axonal transport and synaptic neurotransmission, which require high levels of energy. ATP is produced in mitochondria by the respiratory chain where the electrons’ flow within the complexes generates superoxide anion. This is a free radical resulting from the reaction between oxygen and a small constitutive leak of high-energy electrons. The production of superoxide anion particularly occurs in complexes I and III. Cells possess antioxidant systems such as superoxide dismutase enzyme (SOD) and glutathione, which hinder the production of free radicals. When an imbalance between these opposite strengths occurs, the result is a state defined “oxidative stress,” which indicates an accumulation of excessive ROS and a consequent damage on biological molecules (Morán et al., 2012). Actually, postmortem studies on PD brains showed increased levels of lipid peroxidation, protein carbonyls (Dalfó et al., 2005; Seet et al., 2010), and mtDNA mutations, particularly deletions/rearrangement (Ikebe et al., 1995; Gu et al., 2002; Bender et al., 2006; Dölle et al., 2016) with respect to controls.

In PD, inhibition of respiration, particularly linked to a dysfunction of complex I of respiratory chain, is considered the main source of oxidative stress. Selective deficiency in enzymatic activity of respiratory chain complex I and reduction in glutathione are observed in the SN of PD patients (Schapira et al., 1989, 1990; Mann et al., 1992, 1994; Janetzky et al., 1994), although conflicting results about the evaluation of complex I activity in other PD patients’ tissues such as skeletal muscles, platelets, and leukocytes have been provided (Parker et al., 1989; Yoshino et al., 1992; Didonato et al., 1993; Martín et al., 1996; Winkler-Stuck et al., 2005). Several lines of evidences support the specific involvement of complex I. Neuropathological studies confirmed a reduction in complex I within striatum and SN of PD patients (Mizuno et al., 1989; Hattori et al., 1991). Cybrids, mtDNA-depleted human cells repopulated by mitochondria derived from PD patients and controls, respectively, showed deficient activity of complex I and increased oxidative stress in the former with respect to the latter (Swerdlow et al., 1996; Gu et al., 1998). Especially animal models based on chronic administration of toxins specifically inhibiting complex I of respiratory chain such as 1-methyl-4-phenyl-1,2,3,6-tetrahydropyridine (MPTP), Rotenone and Paraquat (Betarbet et al., 2000; Fornai et al., 2005; Inden et al., 2011; Bové and Perier, 2012) produced pathological and clinical changes akin to PD (Martinez and Greenamyre, 2012). Why complex I is selectively involved remains to be elucidated.

The importance of mitochondrial energy and ROS-associated productions are furthermore highlighted by some autosomal recessive form of hereditary PD whose genes [*PARKIN*, PTEN-induced putative kinase 1 (*PINK1*), and *DJ1*] have a role in mitochondrial bioenergetics and oxidative stress. *PINK1* is a mitochondrial kinase whose deficit was associated to high levels of ROS and more vulnerability to oxidative stress in *Drosophila melanogaster* (Clark et al., 2006) and mouse models (Gautier et al., 2008). Models of human neurons with deficient expression of *PINK1* showed high levels of ROS in mitochondria and cytosol and reduced ATP production (Gandhi et al., 2009). *Parkin* (*PARK2*) encodes for a E3-ubiquitine ligase, which is localized in cytosol but which translocates in mitochondria under stress conditions. Mitochondria with deficit of *Parkin* showed reduced activity of complexes I and IV of the respiratory chain, higher levels of ROS, and more vulnerability to complex I inhibitors (Whitworth et al., 2005). *Parkin*-knockout *Drosophila* model exhibited a similar phenotype to PINK1-mutated flies (Clark et al., 2006), and overexpression of *Parkin* rescues *PINK1*−/− phenotype but not vice versa, suggesting that *PINK1* acts upstream of *Parkin* in a common, linear pathway (Clark et al., 2006; Park et al., 2006; Yang et al., 2006). Fibroblasts from patients affected by *Parkin*-associated PD (Mortiboys et al., 2008) as well as fibroblasts from patients affected by *PINK1*-associated PD (Abramov et al., 2010; Rakovic et al., 2010) exhibited mitochondrial respiratory chain dysfunction and reduced ATP production. *DJ1* is a highly conserved protein encoded by the *PARK7* gene, which likely works as antioxidant. It owns cysteine residues that can be oxidized, and mitochondria isolated from *DJ1*-knockout mice show high ROS levels (Andres-Mateos et al., 2007). Several other functions have been proposed for *DJ1*, however, all linked to oxidative stress management. Indeed, the protein binds, in an oxidation-dependent manner, multiple RNA targets in cells and brain, including mitochondrial genes, genes involved in glutathione metabolism, and members of the phosphatase and tensin homolog (PTEN)/phosphatidylinositol-3-kinase (PI3K) cascade (Van Der Brug et al., 2008). Further, it stabilizes transcription factor Nrf2, which regulates antioxidant response genes (Clements et al., 2006). *DJ1* could also protect cells from stress linked to heavy metals such as copper and mercury acting as a metal binding protein (Björkblom et al., 2013).

The autosomal recessive forms of PD associated to *PINK1*, *Parkin*, and *DJ1* mutations provided evidence of a primary role of mitochondria at least in the pathogenesis of these genetic diseases. However, the role of mitochondria and oxidative stress in sporadic PD remains to be elucidated because the multiple lines of evidence do not allow to discriminate whether impaired mitochondria bioenergetics and ROS-induced biological molecules damages represent a primary pathogenic process or an epiphenomenon of neurodegeneration. A substantial limit to the resolution of this controversy is the fact that it is possible to observe the phenomenon only many years after the occurrence of the primary events. Besides, the absence of Lewy bodies described in the first neuropathological studies performed on *Parkin*-related PD patients threw some doubts as to whether this hereditary form of PD could have the same disease mechanisms as sporadic PD. Nevertheless, nowadays, several studies described the presence of Lewy bodies in *Parkin* brains (Farrer et al., 2001; Pramstaller et al., 2005; Doherty and Hardy, 2013; Miyakawa et al., 2013; Cornejo-Olivas et al., 2015) as well as in two out of three *PINK1* cases (Samaranch et al., 2010; Steele et al., 2015; Takanashi et al., 2016). It has been suggested that this neuropathological heterogeneity could be related to some specific *PINK1* or *PARKIN* mutations and/or to the age of death (Truban et al., 2017).

## Multiple Mitochondria Functions: Evidences From Pink1/Parkin and Other PD-Related Genes

**FIGURE 1:**
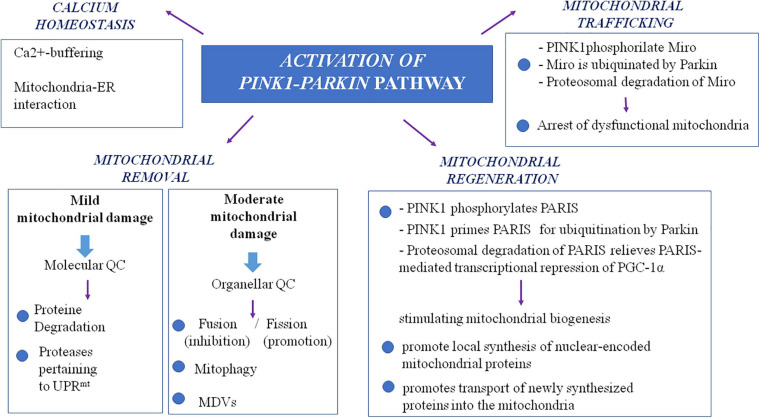
The complex role of PINK1/Parkin pathway in mitochondria. MDVs, mitochondrial-derived vesicles; QC, quality control; PARIS, Parkin interacting substrate; PGC-1alpha, peroxisome proliferator-activated receptor-gamma coactivator; UPRmt, mitochondrial unfolded protein response.

It is noteworthy that a more extensive insight into the autosomal recessive models of PD, particularly *PINK1*- and *Parkin*-associated PD, revealed a wider role of mitochondria in PD pathogenesis, beyond the view of a solely ATP- and ROS-producing organelle (Figure 1). The autosomal recessive models uncovered the importance of multiple mechanisms that maintain healthy mitochondrial pool and then neurons. These mitochondrial pathways are represented by mitochondrial quality control mechanisms, especially mitophagy, mitochondrial trafficking, and mitochondrial calcium homeostasis maintenance. In the next paragraphs, we will try to explain the relationship between PD and these more recently discovered mitochondrial pathways.

### Mitochondrial Quality Control Mechanisms

Studying *PINK1-* and *Parkin*-associated PD, the most frequent autosomal recessive forms of the disease, has been discovered that these two genes are the main responsible of mitochondrial quality control (QC) maintenance, working at various levels. Indeed, they participate both at a molecular level for mild mitochondrial dysfunction and, in a stepwise manner, an organellar level for more severe mitochondrial dysfunction. The former involves molecular chaperones and proteases to correct protein import and folding and to regulate turnover; the latter aims to sequester, sort, and eliminate partially or completely damaged mitochondria. Organellar level involves fusion, fission, and mitophagy processes as well as mitochondrial-derived vesicles (MDVs) pathway.

Mitochondria include over a thousand proteins, the majority of which are encoded by nuclear genome while only approximately 1% of these proteins are encoded by mitochondrial genes. Nuclear-encoded mitochondrial proteins are synthesized in the cytosol and subsequently imported into the organelle by using two complexes localized, respectively, on the outer membrane [translocase of outer membrane (TOM)] and inner membrane [translocase of inner membrane (TIM)] of mitochondria. Inside the organelle, polypeptides fold and assemble into their native functional form these proteins with the involvement particularly of mtHsp60 and mortaline, a member of the Hsp70 family encoded by *HSPA9*. The accuracy of these processes is monitored by chaperones and proteases pertaining to the mitochondrial unfolded-protein response (UPRmt), which is activated by *PINK1*. When proteins are damaged or misfolded, *PINK1* interacts with the serine protease high-temperature-regulated A2 (HtrA2; Plun-Favreau et al., 2007) and the mitochondrial molecular chaperones heat shock protein 75 (Hsp75) also known as tumor necrosis factor (TNF) receptor-associated protein 1 (TRAP1; Pridgeon et al., 2007) promoting their activation by phosphorylation. The relationship between *PINK1* and UPRmt proteases is supported by the finding of a reduction in *HtrA2* phosphorylation in brains of PD patients carrying mutations in *PINK1* (Plun-Favreau et al., 2007). Moreover, brains of *Htra2-*knockout mice exhibit aggregates of misfolded proteins and mitochondrial dysfunction (Moisoi et al., 2009), and these mice develops a phenotype consistent with progressive movement disorder (Jones et al., 2003).

The pathogenic role of this mitochondrial pathway’s dysregulation in PD has further been supported by the finding of mutations in genes involved in UPRmt in PD populations. Thus, in HtrA2 gene, by using a candidate gene approach, a new pathogenic mutation, G399S, has been found in a German cohort of PD patients (Strauss et al., 2005). The same mutation has subsequently been found in a large Turkish family where it cosegregated with PD and essential tremor in a dominant manner (Unal Gulsuner et al., 2014; Chao et al., 2015). Further in the German PD cohort, a polymorphism of HtrA2 gene (A141S) increasing the risk for PD was found. Both G399S and A141S mutations resulted in a deficit of HtrA2 protease activity, and immunohistochemistry and functional analysis in transfected cells showed that both mutation (with the risk allele A141S in a lesser extension) induced changes in mitochondrial morphology and mitochondrial dysfunction (Strauss et al., 2005). Recently, the first mutation in the TRAP1 gene was reported in a patient with late onset PD. The mutation caused complete loss of function of the protein, and functional analysis of patient’s fibroblasts showed increased ROS production associated with increase in mitochondrial biogenesis, damage of UPRmt, reduction in mitochondrial membrane potential (MMP), and sensitivity to mitochondrial apoptosis (Fitzgerald et al., 2017). Furthermore, a missense mutation (c.3614G > A–p.Arg1205His) in eukaryotic translation initiation factor 4 gamma 1 (EIF4G1; *PARK18*) was reported to be pathogenic in a multi-incident northern French family with autosomal dominant, levodopa-responsive, late-onset parkinsonism (Chartier-Harlin et al., 2011). Although doubts on the relevance of *EIF4G1* in the pathogenesis of PD have been risen by the finding of this mutation also in normal controls (Huttenlocher et al., 2015), *EIF4G1* encodes for a transcription factor of mitochondrial proteins that is involved in the UPRmt.

As already highlighted, in the stepwise manner functioning of mitochondrial QC, when deregulation becomes more severe and overwhelms molecular QC, organellar QC processes are involved, and *PINK1/Parkin* axis has a pivotal role also in their promotion and regulation. In healthy mitochondria, PINK1 is imported within mitochondria by TOM and TIM complexes and is sequentially cleaved first at the mitochondrial targeting sequence (MTS) by mitochondrial proteases in the matrix and then at the transmembrane domain (TM) by presenilin-associated rhomboid-like (*PARL*) protease in the internal mitochondrial membrane (IMM; Jin et al., 2010; Deas et al., 2011; Greene et al., 2012). Cleaved *PINK1* is re-exported and degraded by ubiquitin–proteasome system (UPS). In damaged mitochondria, reduced transmembrane potential does not allow PINK1 to enter the organelle; thus, it accumulates on the outer mitochondrial membrane (OMM) in its not cleaved form (Narendra et al., 2010), and it forms a large multimeric complex with TOM (Plun-Favreau et al., 2007; Pridgeon et al., 2007; Narendra et al., 2010; Becker et al., 2012; Lazarou et al., 2012; Costa et al., 2013; Yamano and Youle, 2013; Zhang et al., 2013; Morais et al., 2014). In this way, stabilized *PINK1* was demonstrated to phosphorylate *Parkin* on its serine-65 (S65) residue of ubiquitin-like domain, and in turn, *Parkin* is activated in its enzymatic function, thus translocating to damaged mitochondria. In its activated form, *Parkin* assembles onto OMM ubiquitin protein’s chains, which could be phosphorylated also by *PINK1* itself (Fiesel et al., 2015; Truban et al., 2017). As the results of these events, damaged mitochondria are coated with ubiquitin chains and then embedded in autophagosomes. Here, they are degraded by the autophagy pathway (Okatsu et al., 2010). Therefore, mitophagy is a specialized form of autophagy that manages the turnover of irreversibly damaged and dysfunctional mitochondria and that is mainly regulated by *PINK1/Parkin* pathway. Probably, also F-box only protein 7 (*FBXO7*), whose gene mutations are responsible for a rare autosomal recessive form of early onset atypical PD (*PARK 15*), is involved in the regulation of mitophagy. Actually, this protein, which is a component of a multimeric E3 ubiquitin ligase complex, has been demonstrated to interact with both *PINK1* and *Parkin*, and cells expressing a deficit of *FBXO7* showed reduced translocation of *Parkin* into mitochondria and deficit in mitophagy (Burchell et al., 2013).

Nevertheless, to avoid global dysfunction and maintain mitochondrial integrity, mitochondria can resort to fusion and fission (mitochondrial dynamics), which are regulated again by the *PINK1/Parkin* pathway. These two mechanisms, which are hypothesized to be paired, ensure a healthy population of mitochondria to cells replacing damaged component (mtDNA, proteins and respiratory chain components) of the organelle during the turnover. Mitochondrial fusion, by promoting exchanges with neighboring mitochondria, supplies dysfunctional mitochondria with restored proteins and not-damaged mtDNA, avoiding mitophagy (Twig and Shirihai, 2011). Main proteins participating fusion include three GTPases: mitofusin 1 (Mfn1) and mitofusin 2 (Mfn2) involved in OMM fusion and optic dominant atrophy (OPA1) involved in IMM fusion (Chan, 2006). Mitochondrial fission allows to segregate dysfunctional parts of the mitochondria by generating two mitochondria daughters, and the impaired mitochondria daughter then undergoes mitophagy (Celardo et al., 2014). Particularly, during fission, mitochondria endure a drop of membrane potential, which, beyond a certain level, leads the impaired mitochondria daughter to mitophagy (Twig and Shirihai, 2011). Fission mechanism mainly involves the dynamin-related GTPase protein 1 (Drp1), the mammalian Drp1 homolog dynamin-like protein 1 (DLP1), and the mitochondrial fission 1 protein (Fis1) (Eisner et al., 2018). Taking into account that fusion prevents mitophagy whereas fission promotes it, a correct balance between these two mechanisms is required for an efficient mitophagy and, subsequently, the maintenance of a healthy mitochondria pool. *PINK1/Parkin* pathway is involved in the regulation of mitochondrial dynamics, inhibiting mitochondrial fusion. Indeed, both Mfn1 and Mfn2 are substrate of *PINK1* and *Parkin*; thus, when they are in active state on the OMM, they are ubiquitinated, phosphorylated, and degraded. This event prevents refusion of damaged mitochondria with the healthy network (Chen and Dorn, 2013).

More recently, the *PINK1/Parkin* pathway has been found to be involved also in an alternative organellar QC mechanism for degradation, which prevents global mitophagy: the formation of the so-called MDVs (Truban et al., 2017), a mechanism in which the fission protein Drp1 is not involved (Neuspiel et al., 2008). It has been suggested that a local increase in oxidized proteins and lipids or higher ROS levels that induce a localized reduction of MMP could lead to local *PINK1/Parkin* pathway activation by preventing inactivation of *PINK-1*, which is not imported within mitochondria (Sugiura et al., 2014). This local activation of the *PINK1/Parkin* pathway could lead to the formation of vesicles from mitochondria containing oxidized proteins. These vesicles are directly degraded within the lysosome without the involvement of autophagic machine (Soubannier et al., 2012). Aside from MDVs directed to lysosomes, MDVs directed to peroxisomes have also been identified. Nowadays, only one protein is known to travel by using this type of MDVs, a mitochondrial-anchored protein ligase (MAPL or Mul1) (Braschi et al., 2010). It may likely be important in the regulation of balance between mitochondrial fusion and fission events taking into account that MAPL is responsible for the stabilization of Drp1 and for the degradation of Mfn2, conjointly thus increasing mitochondrial fission (Braschi et al., 2009). Braschi et al. (2010) also showed that binding of MAPL and recruitment of peroxisome MDVs need the involvement of vacuolar protein sorting 35 (VPS35) and vacuolar protein sorting 36 (VPS36), two components of retromer complex that mediates retrograde transport from endosomes to the *trans*-Golgi network. Interestingly, several mutations of the *VPS35* gene were described in PD families with an autosomal dominant inheritance of the disease (*PARK17*; Vilariño-Güell et al., 2011; Zimprich et al., 2011). It is supposed that they cause an imbalance in mitochondrial dynamics by an impairment in fusion with a consequent fragmentation of mitochondria (Tang et al., 2015; Wang et al., 2016). Indeed, functional analysis of *VPS35*-depleted-dopaminergic neurons and neurons expressing D620N mutation, the most frequent in *VPS35*, showed that in these cells, MAPL is not bound anymore, and thus, it has not been degraded. Consequently, increased stabilization of Drp1 and degradation of Mfn2 happen, and fission increases causing fragmentation of mitochondria. Cells expressing D620N mutation in *VPS35* exhibited increased levels of MAPL and decreased Mfn2 levels (Tang et al., 2015). These results suggest the possible role of the dysregulation of fission in pathogenesis of familial and maybe sporadic PD. However, mutations in *VPS35* are rare and explain only 0.2% of sporadic PD cases (Hernandez et al., 2016). The important role of mitochondrial QC in PD pathogenesis is supported also by the finding of mutations in the vacuolar protein sorting 13C (*VPS13C*) gene, which causes an autosomal recessive early onset parkinsonism with rapid progression and early cognitive decline (*PARK 23*; Lesage et al., 2016). Indeed, the *VPS13C* gene encodes for a protein that is partly localized to the outer membrane of mitochondria and whose deficit was associated in cell models with lower mitochondrial membrane potential, mitochondrial fragmentation, exacerbated *PINK1/Parkin*-dependent mitophagy, and transcriptional upregulation of PARK2 in response to mitochondrial damage (Lesage et al., 2016).

The failure of both molecular and organellar levels of mitochondria QC mechanisms leads to irreversible damage of mitochondria and consequent release of its components and proapoptotic proteins (including cytochrome c) into the cytosol. These events, by the apoptotic pathway, lead to cell death (Youle and Strasser, 2008), which could also be strengthened by the activation of neuroinflammatory mechanisms (Hirsch and Hunot, 2009). Indeed, the release into the cytosol of mtDNA together with other compartmentalized mitochondrial molecules could activate a Toll-like receptor (TLR) 9-mediated inflammatory response (Oka et al., 2012), which induces the production of interleukin (IL)-1β and other proinflammatory cytokines (Miura et al., 2010). These events could represent the way by which *PINK1* and *Parkin* mutations provoke loss of dopaminergic neurons and PD.

### Mitochondrial Trafficking

Another important mitochondrial function is their trafficking within the cells, according to local energy demand. It is particularly important in neurons where synapses, which are highly-demanding energy structures, are often located at long distances from cell bodies. Thus, to provide ATP along the entire neuron, mitochondria travel from soma to axons (anterograde axonal transport) through the association with the kinesin family motor proteins KIF1Bα and KIF5, while the transport back to the cell soma (retrograde transport) is mediated by cytosolic dynein (Hollenbeck and Saxton, 2005; Hirokawa et al., 2010). Following studies revealed the presence on the mitochondrial surface of a motor/adaptor complex involved in mitochondrial transport. This complex is formed by the proteins Miro, the heavy chain of kinesin-1 (KHC), Milton, and dynein. Miro is a mitochondrial Rho-like GTPase, which is attached to the OMM and directly interacts with Milton, which in turn recruits the KHC to mitochondria. Thus, the Miro–Milton complex links KHC to mitochondria for anterograde transport of the organelle. Dynein is a cytoplasmic protein mainly involved in retrograde transport by the interaction with kinesin-1 (Stowers et al., 2002; Fransson et al., 2003, 2006; Guo et al., 2005; Hollenbeck and Saxton, 2005; Glater et al., 2006).

It is supposed that impairment in mitochondria trafficking could have a role in the pathogenesis of PD. Indeed, *PINK1* and *Parkin* have been found to be associated with the Miro/Milton complex, suggesting their involvement in mitochondrial trafficking (Weihofen et al., 2009; Wang et al., 2011). *PINK1* is able to phosphorylate Miro on multiple sites, and this event promotes ubiquitination by *Parkin*. This results in proteosomal degradation of Miro and lacking formation on mitochondria surface of the complex, which is essential for the movement of the organelle along the axons. This interaction between *PINK1/Parkin* and Miro is important because it arrests dysfunctional mitochondria, preventing corruption of healthy mitochondria via fusion and the spreading of ROS within neurons. Thus, it is conceivable that *PINK1* and *Parkin* mutations, producing a loss of function of these proteins, could promote dopaminergic cells’ death and PD also by an impairment of mitochondrial trafficking. In line with this observation, *Drosophila* model of PD expressing a deficit of *PINK1* exhibited mitochondrial transport defects (Liu et al., 2012). To strengthen the possible role of altered mitochondria trafficking in PD pathogenesis, the protein leucine-rich repeat kinase 2 (*LRRK2*), which is involved in the most frequent autosomal dominant form of PD, has been demonstrated to interact with Miro1 in human-induced pluripotent stem cell (iPSC)-derived neurons. It forms a complex with Miro 1 and removes it from damaged mitochondria, stopping them and preventing their motion along the axons. In this model, the most common pathogenic mutation of *LRRK2* gene, G2019S, suppresses this function of arresting mitochondria, thus slowing the initiation of mitophagy (Hsieh et al., 2016).

Taking into account these observations, *RhoT1*, the gene that encodes for Miro1, has been proposed as a candidate gene for PD. However, to date, it has not found any significant association between polymorphisms of this gene and PD (Anvret, 2012).

### Mitochondrial Calcium Homeostasis

Mitochondria are also involved in calcium homeostasis, which is critical for appropriate neuronal functioning considering that calcium signal regulates several processes including not only synaptic release of neurotransmitters and vesicle recycling but also neuronal plasticity and axonal transport. Mitochondria represent an important buffer for Ca^2+^ as demonstrated by the rapid increase in cytosolic Ca^2+^ concentration after stimulation of these organelles with specific agonists. Thus, mitochondria allow the accumulation of high levels of Ca^2+^ in specific subcellular domain as observed in neurons where, for example, they localize in the synaptic terminal near Ca^2+^ channels to accumulate this ion and prevent its spreading (Rizzuto et al., 2012). Mitochondria also present tight contacts with endoplasmic reticulum (ER), which is the main calcium stock in cells. This Ca^2+^-buffering function of mitochondria is particularly important in dopaminergic neurons of the substantia nigra, which have an activity of autonomous pacemaker in order to allow the sustained release of dopamine. As L-type calcium channels are essential for this mechanism, these neurons are exposed to high fluxes of Ca^2+^ (Surmeier et al., 2011). Dopaminergic neurons need higher levels of ATP to maintain adequate cytosolic calcium concentration. Deregulation of calcium homeostasis with mitochondrial calcium overload could lead to dramatic consequences for cells, such as apoptosis and death. A link between altered calcium homeostasis and PD has been suggested by the finding of mitochondrial calcium overload in Rotenone models of PD (Yadava and Nicholls, 2007) as well as in *PINK1*-deficient neurons where excess of mitochondrial calcium leads to apoptotic cell death (Gandhi et al., 2009). Furthermore, some authors have demonstrated that *α-syn*, *Parkin*, and *DJ1* are important for the ER–mitochondria interaction aimed to the transfer of calcium (Calì et al., 2012a, b). Therefore, mutations in the genes encoding for these proteins, involved in inherited forms of PD, could promote pathogenesis of the disease also through abnormal mitochondrial calcium accumulation.

### Mitochondrial Biogenesis

Another aspect of mitochondrial functions, which could be involved in PD pathogenesis, is mitochondrial biogenesis, as demonstrated by *PINK1/Parkin* pathway role also in this field. In the dopaminergic neurons of substantia nigra, a Krüppel-associated box domain zinc finger protein (KRAB-ZFP) has been identified and denominated Parkin-interacting substrate (PARIS). Its name is explained by the fact that the expression of PARIS is regulated by Parkin via the UPS. Indeed, *in vitro Parkin* is able to ubiquitylate the transcriptional repressor PARIS, inducing its proteasomal degradation (Shin et al., 2011). A known function of PARIS is to repress the expression of the peroxisome proliferator-activated receptor gamma (PPARγ) coactivator-1α (PGC-1α) (Shin et al., 2011), which is an important inducible coactivator of nuclear receptor. PGC-1α stimulates mitochondrial biogenesis through the induction of expression of nuclear respiratory factors and mitochondrial transcription factor A, a regulator of mtDNA replication and/or transcription (Wu et al., 1999). Therefore, loss of function mutations in *Parkin* gene could promote cell death by impairing mitochondrial biogenesis (Stevens et al., 2015). Indeed loss of Parkin could induce overexpression of PARIS with consequent excessive repression of PGC-1α.

**FIGURE 2:**
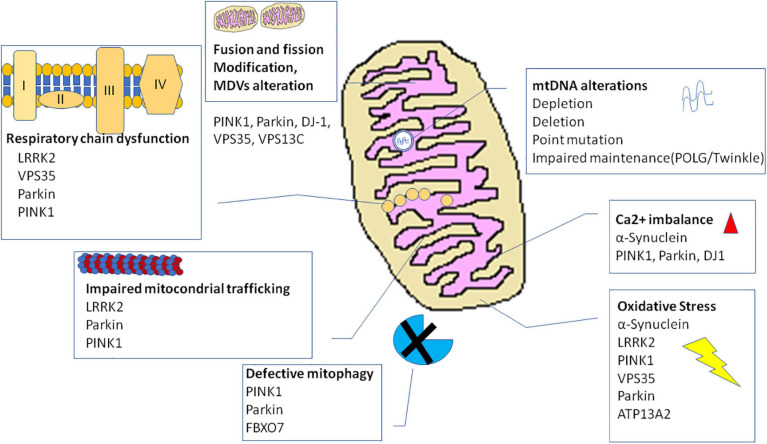
Mechanisms and genes involved in mitochondrial dysfunction in Parkinson’s disease. ATP13A2, ATPase cation transporting 13A2; DJ-1, protein deglycase; LRRK2, leucine-rich repeat kinase 2; *PINK1*, phosphatase and tensin homolog (PTEN)-induced putative kinase 1; VPS35, vacuolar protein sorting 35.

The many evidence explained thus far describe an involvement of mitochondria in PD pathogenesis at multiple levels (for summary, see Table 1 and Figure 2). Several mitochondria insults, such as toxins, aging, and gene mutations, could promote mitochondrial dysfunction by increasing oxidative stress and impairing energy production, dysregulating calcium homeostasis and mitochondrial trafficking, and inducing apoptosis as the result of abnormal QC mechanisms and mitochondrial biogenesis. Nonetheless, recently, within the neurodegenerative process, high consequence has been ascribed to the interaction between mitochondria and amyloidogenic proteins, i.e., α-syn in PD pathogenesis.

**TABLE 1:** Multiple mechanisms underlying mitochondrial dysfunction in the pathogenesis of PD.

Mitochondrial dysfunction	Molecular mechanisms involved	Effects	PD genes potentially involved
Impairment in bioenergetics	Mitochondrial respiratory chain (especially inhibition of *complex I*)	• Reduced ATP production; • Oxidative stress	PARKIN, PINK1, DJ1
Impairment in quality control mechanisms	• UPRmt (e.g., *mtHsp60, mortaline, HtrA2, TRAP1*); • Mitochondrial dynamics: fusion (*proteins Mfn1 andMfn2, OPA1*) and fission (proteins *Drp1, DLP1, Fis1*); • Mitophagy (*mitochondrial proteases in matrix, PARL, Ubiquitin*); • MDVs (*directed tolysosomes and peroxisomes, MAPL or Mul1 protein*)	• Altered proteostasis with accumulation of damaged proteins; • Fragmentation of mitochondria; • Altered turnover, accumulation of dysfunctional mitochondria	PARKIN, PINK1, FBXO7, VPS35, VPS13C, *EIF4G1*
Impairment in Mitochondrial trafficking	Altered axonal transport (involvement of complex *Miro/KHC/Milton/dynei n*)	Unmeet local demands for energy, calcium, redox balance, and other mitochondrial functions	PINK1, PARKIN, LRRK2
Impairment in calcium homeostasis	Mitochondrial calcium overload	• Mitochondrial permeability transition pore opening (e.g., with cytochrome c release); • Oxidative stress	PINK1, α-syn, Parkin, DJ1
Impairment in mitochondrial biogenesis	Reduced synthesis of new mtDNA, protein, and membrane (involvement of *PARIS, PGC1α*)	Imbalance of mitochondrial health, apoptosis	PINK1, Parkin

*α-syn, alpha-synuclein; ATP, adenosine triphosphate; Drpl, dynamin-related GTPase protein 1; DLP1, dynamin like protein 1; Fisl, mitochondrial fission 1 protein; HtrA2, serine protease high-temperature-regulated A2; KHC, kinesin heavy chain; MAPL or Mull, mitochondrial-anchored protein ligase; MDVs, mitochondria-derived vesicles; Mfnl, mitofusinl; Mfn2, mitofusin2; mtDNA, mitochondrial DNA; OPA, optic atrophy protein; PARIS, PARkin interacting substrate; PD, Parkinson’s disease; PGCla, peroxisome proliferator-activated receptor gamma coactivator-la; TRAP1, TNF receptor-associated protein 1.*

## Relationship Between Mitochondrial Dysfunction, α-Syn Aggregates, and Lysosomal System in PD

Considering the role of mitochondria in the pathogenesis of PD, it is necessary to evaluate the relationship between this organelle and α-syn, which represents the pathological hallmark of PD. Indeed, this protein represents the principal constituent of Lewy bodies, and the point mutation A53T in its gene (*SNCA*) was the first to be identified in 1997 in hereditary PD (Polymeropoulos et al., 1997). Alpha-syn is a 140-amino-acid long protein that is mainly located in the presynaptic terminal of neurons where it is involved in synaptic vesicle turnover and endocytosis. Particularly, it is involved into the synthesis, storage, and release of dopamine within neurons of substantia nigra (Yavich et al., 2004).

**FIGURE 3:**
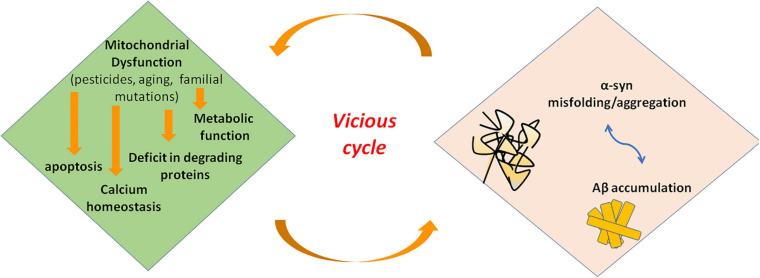
Interaction of misfolded proteins and mitochondria in neurodegenerative disorders. Mitochondrial dysfunction leads to deficit in metabolic function and calcium homeostasis, apoptosis, and possibly impairment of the ability of mitochondria to degrade and import proteins. Amyloidogenic proteins such α-syn and Aβ accumulate in cytosol and aggregate, preventing also mitochondrial import of other proteins. These events increase mitochondrial dysfunction in a vicious cycle. α-syn, alpha-synuclein; Aβ, amyloid beta; ROS, reactive oxygen species.

Mitochondrial dysfunction and α-syn aggregates are linked by a bidirectional relationship, where the former can promote the production of pathological α-syn oligomers, and the latter can cause mitochondrial impairment. However, it is hard to distinguish the primary event in PD (Figure 3). On the one side, several studies demonstrated that excess of ROS production, for instance because of mitochondrial insults, promotes intracellular α-syn misfolding and aggregation (Perfeito et al., 2014; Ganguly et al., 2017). On the other side, wild-type α-syn in high concentration or mutated α-syn tends to acquire a cross-beta amyloid conformation and self-aggregate thus forming oligomers. Larger oligomers are highly toxic because of their greater stability (Parnetti et al., 2014; Pieri et al., 2016) and can impair multiple cellular functions including mitochondrial energy production. Experimental evidence suggest an interaction of α-syn with complex I of respiratory chain. Indeed, in cell models overexpressing α-syn, oxidative stress and mitochondria changes were associated to depolarization of mitochondrial membrane and decreased activity of complex I of mitochondrial respiratory chain (Hsu et al., 2000). Thus, an inhibitory effect on complex I has be suggested for α-syn that could act in a dose-dependent manner as reported in a study on rat brains (Liu G. et al., 2009). Besides *α-syn*, knockout mice are resistant to the effects of MPTP, suggesting that α-syn is required downstream of complex I (Dauer et al., 2002).

More recently, a new understanding of the role of mitochondrial dysfunction in the pathogenesis of neurodegenerative disease has been proposed. Indeed, these organelles could directly be involved in importing and degrading amyloidogenic proteins such as α-syn (Lautenschäger and Schierle, 2019). Many evidence showed that α-syn can tie OMM. Particularly, an interaction with Tom 20 and Tom 40, proteins pertaining to TOM complex, has been demonstrated (Di Maio et al., 2016; Martínez et al., 2018; Ryan et al., 2018). Alpha-syn has also been demonstrated to interact with ATP synthase, thus suggesting a localization of this protein at the IMM (Ludtmann et al., 2016). In line with these evidence, α-syn includes in its N-terminus domain an amino acidic sequence similar to those with mitochondrial targeting properties (Miraglia et al., 2018). Changes into the N-terminus of α-syn influence the affinity for lipids, thus modifying the ability of the protein to bind mitochondrial membranes and conditioning the subcellular localization of α-syn (Lin et al., 2019). Particularly, α-syn derived from point mutation A53T of *SNCA* gene exhibit high affinity for mitochondrial membrane, and it is supposed that α-syn could influence mitochondrial size and shape. Actually, the analysis of human-derived neurons expressing A53T- α-syn showed a higher fraction of this protein localized into mitochondria as well as mitochondria fragmentation and defect in mitochondrial transport (Pozo Devoto et al., 2017). A role of mitochondria in degrading α-syn aggregates is supported also by the demonstration of an interaction between the mitochondrial protease HtrA2 and α-syn (Liu M.L. et al., 2009). Besides, in yeast, protein aggregates formed under heat shock conditions are imported into mitochondria for degradation (Ruan et al., 2017). Some authors proposed that α-syn enters mitochondria to perform a specific and physiological activity. Actually, a study performed on mice brains showed that monomeric α-syn modulates ATP synthase function by interacting with its α subunit (Ludtmann et al., 2016). Alpha-syn aggregates could also obstruct the mitochondrial import protein system preventing the import of other proteins important for proper functioning of mitochondria. This fact could interfere with mitophagy preventing proper activation of PINK1/Parkin pathway. Thus, proteins involved in genetic forms of PD could also participate the pathogenesis of the sporadic form of disease (Di Maio et al., 2016). However, further investigations are required. Taking into account these observations, Lautenschäger and Schierle (2019) speculated that mitochondrial insults could impair the ability of mitochondria to degrade amyloidogenic proteins. The deficit in α-syn degradation could lead to an increase in the cytosolic levels of protein that tend to aggregate. An impairment of import and degradation of physiologically relevant mitochondrial proteins could also occur, thus intensifying mitochondrial dysfunction (Lautenschäger and Schierle, 2019).

Alpha-syn has also been demonstrated to interact within the interconnection between ER and mitochondria, which are essential to neuron survival (Lin et al., 2019). In stress conditions, ER transfers high levels of Ca^2+^ to the mitochondria, increasing the production of ROS (Melo et al., 2018).

It has been proposed that dopaminergic neurons are particularly vulnerable to the formation of highly toxic α-syn oligomers considering their high density of synapses and above all the increased oxidative stress related to dopamine production. The high ROS production, which is also increased by the oxidized dopamine, further promotes the aggregation of α-syn and its consequent accumulation in Lewy bodies in a vicious cycle manner (Mor et al., 2017).

Furthermore, the relationship between α-syn and monoamine oxidase-B (MAO-B), which is an outer mitochondrial membrane protein that oxidizes arylalkylamine neurotransmitters, is thought to be significant in the degeneration of dopaminergic neurons (Kang et al., 2018). The expression of MAO-B increases with aging and in neurodegenerative processes, and it is associated with higher levels of ROS and free radical damage (Mahy et al., 2000). Recently, the metabolite produced by the enzymatic activity of MAO-B on dopamine, 3,4-dihydroxyphenyl acetaldehyde (DOPAL), has been demonstrated to eventually further stimulate enzymatic activity of MAO-B itself by triggering the activity of asparagine endopeptidase (AEP or leguminase). This enzyme cleaves α-syn at its residue N103 producing a fragment (α-syn 1–103) that binds MAO-B and stimulates its enzymatic activity, more efficiently than α-syn wild type. The consequence is an increased activation of MAO-B and an enhanced production of the neurotoxic methabolite DOPAL in a vicious cycle leading to dopaminergic neurodegeneration. Virally mediated expression of α-syn 1–103 induces PD pathological changes but only in mice expressing MAO-B, underlining the importance of this mitochondrial enzyme in PD pathogenesis (Kang et al., 2018).

It is also important to consider that an excess of α-syn as well as an impairment in mitochondrial function could also be promoted by a dysfunction of one or both the main proteolytic pathways in cells, the UPS and the lysosome system, the latter including macroautophagy (or simply autophagy), chaperon-mediated autophagy (CMA), and endocytosis (Rott et al., 2017; Grassi et al., 2018; Limanaqi et al., 2019). All of these autophagy routes converge on the only lysosome that is involved in proteostasis and degradation of several classes of macromolecules. This function is particularly important in long-lived, postmitotic cells such as neurons. An impairment at each level of this network can lead to insufficient clearance of pathogenic proteins, defective membrane trafficking and signaling, and cell damage (Nixon, 2020). Misfolded proteins are targeted for protease degradation, including toxic forms of α-syn. These, as well as non-toxic monomers, are degraded through macroautophagy. However, a dysfunction in these pathways could cause a reduced removal of damaged wild-type proteins such as α-syn (or mutated proteins), which accumulates in degrading organelles. Thus, toxic species damage proteolytic pathways in a vicious cycle manner. According to this hypothesis, mutations in genes involved in lysosomal system have been identified in several forms of PD, such as *ATP13A2* and *GBA*, the gene encoding for glucocerebrosidase 1, a hydrolase that catalyzes the metabolism of glucosylceramide and whose loss of function represents one of the main genetic risk factor for PD (Aharon-Peretz et al., 2004). Further, patients affected by lysosomal storage diseases with mutations in lysosome system genes can exhibit parkinsonism as a clinical feature (Stamelou et al., 2013). Recently, also the gene leucine-rich repeat kinase 2 (LRRK2) has been linked to lysosomal function. Mutations in this gene represent one of the most frequent genetic cause of PD, and they are found in 1–2% of sporadic PD cases (Healy et al., 2008). Despite that, the function of the protein encoded by this gene remains unclear, with evidence for a role in synaptic transmission, endolysosomal trafficking, cellular proliferation, and cytoskeleton dynamics. A recent study (Ysselstein et al., 2019) showed that mutations in LRRK2, which gain the kinase activity of the encoded protein, act as negative regulator of the lysosomal glucocerebrosidase-1 activity. The augmentation of LRRK2 kinase activity could reduce the glucocereborsidase-1 activity by increasing the level of phosphorylated Rab10, a GTPase potentially involved in endolysosomal function and lysosomal homeostasis, which is demonstrated to be an LRRK2 substrate (Eguchi et al., 2018). Interestingly, increased LRRK2 kinase activity was observed in idiopathic PD, suggesting a possible role of this gene in sporadic PD pathogenesis (Di Maio et al., 2018).

Nonetheless, mutated species of α-syn could primarily impair UPS and/or lysosome system. Several authors suggest a trilateral relationship of mitochondrial dysfunction, α-syn aggregation, and degradation of the endolysosomal and proteasome systems in the neurodegenerative process of PD (Pozo Devoto and Falzone, 2017; Grassi et al., 2018; Vicario et al., 2018). Both mitochondria dysfunction and endolysosomal impairment are involved in the development of α-syn pathology and vice versa. An intricate interplay is supposed, in which dysregulation of one system influences the proper activity of the other two. However, which is the starting factor is not known. They could interact with each other simultaneously in promoting PD process, or most likely, in each patient, the interaction between the genetic equipment and environmental factors defines the most involved system and so the initiating factor. This is supported also by the high variability in clinical presentations of PD. Then, a vicious cycle could develop once one system becomes dysfunctional (Lin et al., 2019).

## Mitochondrial Monogenic Parkinsonisms

Since the first discovery of primary mtDNA mutations in 1988, only a few case reports have linked mtDNA mutations with parkinsonism. These include m.11778G > A in MT-ND6, a heteroplasmic 4 bp deletion in MT-CYB21, and m.8344A > G in MT-TK (Giannoccaro et al., 2017). Furthermore, parkinsonism has not been observed in several national cohort studies of common mtDNA mutations (Mancuso et al., 2013, 2014; Nesbitt et al., 2013), suggesting that the association with primary mtDNA mutations is more likely to be an incidental co-occurrence. We have also failed to demonstrate the connection of m.8344A > G mutation in 159 patients with PD (Mancuso et al., 2008). Moreover, neuropathological studies have not identified significant mtDNA mutations in the substantia nigra of patients with idiopathic PD (IPD) compared to controls. Based on these findings, we believe that primary mtDNA point mutations do not play a role in the pathogenesis of PD.

However, while parkinsonism is an extremely rare clinical feature of a mitochondrial disorder caused by a primary mitochondrial DNA mutation, in those mitochondrial diseases determined by mutations in nuclear genes involved in mtDNA replication and maintenance, parkinsonism is quite a common clinical feature, usually associated with mitochondrial myopathy and chronic progressive external ophthalmoplegia (CPEO) (Giannoccaro et al., 2017). In a large, national cohort United Kingdom study, parkinsonism accounted for 43% of all extrapyramidal movement disorders in patients with mitochondrial disease, making the most common extrapyramidal movement disorders identified in this population. The most common genetic defect associated with parkinsonism is POLG mutations (Martikainen et al., 2016).

## Mitochondrial Dysfunction in Atypical Parkinsonisms

The role of mitochondrial dysfunction in the pathogenesis of atypical parkinsonism has been recently investigated. The involvement of mitochondria in pathogenetic pathways is demonstrated in both synucleinopathies and tauopathies. Multiple system atrophy (MSA), a synucleinopathy together with PD and dementia with Lewy bodies, is a neurodegenerative disorder in which a variable degree of parkinsonism, cerebellar ataxia, dysautonomia, and pyramidal features coexist. Considering the predominant symptomatology, parkinsonian (MSA-P) or cerebellar (MSA-C) subtype has been described (Fanciulli and Wenning, 2015; Krismer and Wenning, 2017). In neuropathological studies, MSA shows an accumulation of α-syn in neurons and oligodendrocytes (Jellinger, 2018). Although the aberrant protein localization is known for many years, pathogenic mechanisms are almost unclear, and several processes such as α-syn overexpression, cell-to-cell transfer, inflammation, and mitochondrial functioning have been proposed (Jellinger, 2018). A reduced complex I activity in MSA patients’ skeletal muscle (Blin et al., 1994), but not in platelets or SN (Gu et al., 1997), has been observed. Additionally, an impairment of enzymatic activities related to respiratory chain complex II in fibroblast primary cultures of MSA patients has also been demonstrated (Monzio Compagnoni et al., 2018a).

Recently, an increasing interest has been drawn to coenzyme Q2, polyprenyltransferase (COQ2). COQ2 is involved in mitochondrial respiratory chain, playing an important role in transferring electrons from complexes I and II to complex III (Monzio Compagnoni et al., 2020). Mutations of COQ2 gene are thought to have a causative role in familial and sporadic cases of MSA (Mitsui et al., 2013). Recessive COQ2 mutations cause primary CoQ10 deficiency, leading to an infantile encephalomyopathy/nephropathy with cerebellar atrophy. Similarly, other primary CoQ10 deficiency syndromes are characterized by cerebellar ataxia as the main clinical feature (Quinzii et al., 2006; Schottlaender and Houlden, 2014). However, it is still controversial whether variants in the gene encoding COQ2 increase the risk of MSA (Jeon et al., 2014; Schottlaender and Houlden, 2014; Ronchi et al., 2016). Although conflicting results are available about the role of COQ2 mutations in MSA, a reduced CoQ10 amount has been observed in the MSA cerebellum (Barca et al., 2016; Schottlaender et al., 2016), cerebrospinal fluid (Compta et al., 2018), plasma (Mitsui and Tsuji, 2016), serum (Kasai et al., 2016), and fibroblasts (Monzio Compagnoni et al., 2018a), independently from COQ2 mutational status. Nevertheless, in MSA brains, a reduction in two enzymes involved in CoQ10 synthesis (decaprenyl diphosphate synthase subunit 1—PDSS1 and coenzyme Q5, methyltransferase—COQ5) has been found (Barca et al., 2016; Monzio Compagnoni et al., 2018b). A recent interesting study demonstrated an impairment in autophagy and mitochondrial functioning in MSA neurons (Monzio Compagnoni et al., 2018b). In particular, the authors not only identified an impaired activity of respiratory chain complexes (specifically complex II and complexes II + III), but they also observed an increase in mitochondrial mass and the upregulation of several enzymes involved in multiple mitochondrial pathways, including the CoQ10 synthesis, suggesting that it might be related to a mitochondrial attempt to compensate the functional deficit (Monzio Compagnoni et al., 2018b). Although data about the role of mitochondrial dysfunction in the pathogenesis of MSA are encouraging, it is still unclear whether it is a cause or a consequence of misfolded proteins accumulation. Up to now, how mitochondrial dysfunction and the α-syn accumulation in MSA are related is not completely clarified.

Evidence from laboratory and *in vivo* studies suggest a mitochondrial dysfunction also in progressive supranuclear palsy (PSP). A reduction in complex I activity in PSP cybrid lines, which are transmitochondrial cytoplasmic hybrid (cybrid) cell lines expressing mitochondrial genes from patients with PSP, was demonstrated several years ago (Swerdlow et al., 2000). Using the same techniques, increased activity of antioxidant enzymes and oxidative damage to lipids was revealed (Swerdlow et al., 2000; Albers et al., 2001; Chirichigno et al., 2002). Moreover, the presence of oxidative stress in PSP brains is found in postmortem immunochemical studies (Dexter et al., 1992; Sian et al., 1994; Jenner and Olanow, 1996; Odetti et al., 2000; Cantuti-Castelvetri et al., 2002). A reduction in high-energy metabolites in the brains of PSP patients was also shown using combined phosphorus and proton magnetic resonance spectroscopy (Stamelou et al., 2009). In this study, there was a decreased concentration of high-energy phosphates (=ATP + phosphorylated creatine) and inorganic phosphate in the basal ganglia of PSP patients, whereas no significant differences in low-energy phosphates (=ADP + unphosphorylated creatine) were found in comparison to controls. The frontal lobe, but not the occipital lobe, showed similar alterations. A peak of lactate, which is related to increased anaerobic glycolysis, was found in 35% of PSP patients (Stamelou et al., 2009).

An interesting association was found in the French West Indies between atypical parkinsonism and the habitual intake of *Annona muricata*, which is a plant belonging to the Annonaceae family. About one-third of the affected patients developed a PSP-like parkinsonism (Lannuzel et al., 2007). Annonaceous plants produce Annonaceous acetogenins, a family of lipophilic complex I inhibitors (Ries et al., 2011). The major acetogenin contained in *A. muricata* is annonacin. It has been demonstrated to be ∼1,000 times more toxic to cultured mesencephalic neurons than 1-methyl-4- phenylpyridinium (MPP+) (A. Lannuzel et al., 2003). In rats treated with chronic systemic infusion of annonacin, reduced brain ATP levels, along with neuronal cell loss and gliosis in the brainstem and basal ganglia reflecting a PSP-like pattern, were found (Champy et al., 2004). Moreover, a redistribution of tau from the axons to the cell body is induced by annonacin, leading to cell death. Further compounds to which humans are potentially exposed are able to inhibit the complex I of the mitochondrial chain. They were also demonstrated to decrease ATP levels, induce neuronal cell death, and cause the redistribution of tau from axons to the cell body; therefore, their potency in inhibiting the complex I correlated with their potency in inducing tau redistribution (Escobar-Khondiker et al., 2007; Höllerhage et al., 2009). A loss of neurons in the SN and in the striatum, associated with abnormally high levels of tau immunoreactivity in the cytoplasm of neurons, oligodendrocytes, and astrocytes, was demonstrated in rats treated with rotenone (complex I inhibitor) (Höglinger et al., 2005). The toxicity of rotenone is attenuated by CoQ10, which preserves the mitochondrial membrane potential in cultured neurons (Menke et al., 2003; Moon et al., 2005). These preliminary results led to investigating the effect of CoQ10 on PSP. However, although a short-term effect of CoQ10 in 21 clinically probable PSP patients was found in terms of a mild clinical improvement along with significant increase in cerebral energy metabolism (Stamelou et al., 2008), a following 12-month study on 61 PSP patients using high doses of CoQ10 did not significantly improve PSP symptoms or affect disease progression (Apetauerova et al., 2016).

## Mitochondrial Dysfunction in Huntington’s Disease

Mitochondrial dysfunction has been reported also in Huntington’s disease (HD), and mitochondrial defects could be involved in the region-specific pattern of HD degeneration (Damiano et al., 2010). HD is an autosomal-dominant inherited progressive and eventually fatal neurodegenerative disease, the typical manifestations of which are involuntary movements, psychiatric symptoms, and cognitive decline (Walker, 2007). The etiological basis is the deleterious expansion of polyglutamine (PoliQ) encoding CAG repeats in the exon 1 of the huntingtin (*HTT*) gene, leading to the expression of neurotoxic mutant huntingtin (mHTT) (MacDonald et al., 1993). The disease usually starts in midlife, with age of onset inversely correlating to CAG repeat number. The greater the number of CAG repeats, the earlier the age of onset, and the greater the severity of the disorder (Tabrizi et al., 2013). Extensive degeneration of neurons primarily occurs in the striatum and cortex. Striatal medium spiny GABAergic neurons (MSNs) are the most vulnerable in front of a relative sparing of the large striatal neurons, including the striatal interneurons. Moreover, different degrees of degeneration could be also observed within the striatal neuronal population with a more severe involvement of the indirect pathway expressing predominantly D2 receptors (Vonsattel and DiFiglia, 1998). Although the HD mutation has been identified, the molecular processes that determine HD pathogenesis are not yet fully understood. Several lines of evidence indicate that the CAG expansion predominantly leads HTT to gain a toxic function (Cisbani and Cicchetti, 2012). However, HTT is a cytoplasmic protein expressed widely throughout the body but with the highest expression in the brain and testes, which has been shown to interact with a wide variety of transcription factors and to serve as a scaffold to coordinate complexes of other proteins. As a result, its depletion seems to disrupt several processes that are fundamental for the survival and functioning of the neuron, including endocytosis, vesicle trafficking, RNA biogenesis, endocytosis, mitosis, transcriptional regulation, postsynaptic signaling, apoptotic signaling pathway, and defects in energy metabolism (Ross and Tabrizi, 2011). Additionally, the resultant longer polyQ tracts of HTT are prone to aggregate with ubiquitin-positive proteins, which are a pathological hallmark of HD. Overall, decades of intense research using cell models, animal models, and postmortem HD brains have implicated a critical role of mitochondrial dysfunction in HD progression and pathogenesis (Yan et al., 2020). The point at which mitochondrial involvement begins is unclear, but there is evidence that mitochondrial impairment occurs even in asymptomatic HD carriers (Saft et al., 2005). In HD, dysfunctional mitochondria have been shown to trigger both neuronal apoptosis and necrosis, disrupt glia, and initiate the inflammatory cascade (Lin and Beal, 2006). Other crucial cellular changes involved in HD pathogenesis, including *N*-methyl-D-aspartate receptor (NMDAR) activation, caspase activation, calcium dyshomeostasis, and abnormal axonal trafficking, require a normal mitochondrial function. It has been suggested that mHTT could have direct or indirect effects on mitochondria, compromising energy metabolism and increasing oxidative damage. There is extensive indirect evidence for bioenergetic deficits in HD, such as a body weight loss despite sustained caloric intake, nuclear magnetic resonance spectroscopy showing increased lactate in the cerebral cortex and basal ganglia, and defective cerebral glucose metabolism in PET studies of the brains of HD patients (Reddy and Shirendeb, 2012). Postmortem striatum samples of HD patients showed first a reduced activity of mitochondrial complexes II–IV of the electron-transport chain and aconitase (Gu et al., 1996; Browne et al., 1997), correlating with reduced levels of ATP in the mutant neurons and reduced uptake of substrates by mitochondria. Later, studies in the brain tissue of HD transgenic and knock-in mice confirmed a decreased activity of complexes I–IV (Pandey et al., 2008). In keeping with this, mitochondrial toxins that selectively inhibit succinate dehydrogenase and complex II, such as rotenone and 3-nitropropionic acid, induce a clinical and pathological phenotype that closely resembles HD (Beal et al., 1993). Notably, cellular energy metabolism seems to be impacted extremely early in the cascade of HD pathogenic events (Browne, 2008). Several authors argued that striatal neurons are particularly sensitive to defects of the mitochondrial oxidative phosphorylation due to their high-energy demand (Pickrell et al., 2011). However, mitochondrial alterations in HD result from a combination of disease-promoting pathways. mHTT associates with the outer mitochondrial membrane in different HD models, resulting in mitochondrial membrane potential loss, cytochrome c release, protein import deficit, and increased sensitivity to calcium-induced mitochondria permeabilization (Bossy-Wetzel et al., 2008; Lautenschäger and Schierle, 2019). Their major function is energy metabolism, but they also play an important role in buffering and shaping cytosolic calcium rises and in mediating cell death by apoptosis. Furthermore, mitochondrial dysfunction could result in overproduction of ROS and nitrogen-reactive species (RNS) and/or failure of the antioxidant defense leading to oxidative/nitrative stress, which is associated with HD. Oxidative damage is fluid (Stack et al., 2008). mtDNA is a major target of the oxidative stress associated with mHTT. Accordingly, higher frequencies of mtDNA deletions were found in HD patients than healthy controls (Banoei et al., 2007). Mitochondrial loss and altered mitochondrial morphology and dynamics have been observed in HD brain and worsen with increasing disease severity (Kim et al., 2010). The balance between fission and fusion is important for maintaining normal mitochondrial function. High levels of fission genes (e.g., *Drp1*), low levels of fusion genes (e.g., Mfn1), and high levels of cyclophilin D have been selectively found in striatum and cortex specimens from HD patients (Shirendeb et al., 2011). Specifically, induction of mitogen-activated protein kinase 1 (MAPK1) can upregulate Drp1 activity causing mitochondrial fragmentation (Roe and Qi, 2018). Additionally, mHTT also translocates to the nucleus, where it binds and increases the level and transcriptional activity of p53, which interacts with Drp1 leading to mitochondria fragmentation (Guo et al., 2014). Recently, Zhao et al. (2019) have found in HD that mitochondrial protein ATPase family AAA-domain containing protein 3A (ATAD3A), an interactor of Drp1, exhibits a gain of function that causes mitochondrial fragmentation and impairs mitochondrial biogenesis. A new peptide inhibitor has also been developed to decrease Drp1 interaction with ATAD3A suppressing mitochondrial fragmentation and mtDNA damage, as well as reducing HD neuropathology (Zhao et al., 2019). Finally, in HD neurons, the increase in mitochondrial free radicals activates another fission protein called Fis1, which promotes an increase in mitochondrial fragmentation. Both *PINK* and *parkin* are involved in mitophagy, and previous studies in *Drosophila* models of HD displayed that *PINK1* overexpression can affect the efficiency of the mitophagy process by inhibiting mHTT activity (Khalil et al., 2015). mHTT has been also found to activate autophagy by inhibiting the mechanistic target of rapamycin (mTOR) (Lee et al., 2015). Expression of PGC-1α, which provides neuroprotective effects by activating autophagy and is a coregulator of mitochondrial biogenesis and antioxidant enzymes (as seen in PD), is reduced in HD, contributing to mitochondrial impairment (Johri et al., 2013). Indeed, mice knockout for *PGC-1α* shows a clinical phenotype similar to HD (Lin et al., 2004). As mentioned, mHTT demonstrated to directly interact with various cellular proteins, and the stress-responsive transcription factor (HSF1) has been reported as the major transcriptional regulator factor impaired in HD (Gomez-Pastor et al., 2017). Recently, Intihar et al. (2019) proposed the existence of alterations in a common p53-HSF1-PGC-1α axis in mediating transcriptional dysregulation and mitochondrial dysfunction in HD. Additionally, the association of the valosin-containing protein (VCP), a multifunctional protein implicated in protein degradation, with mHTT at mitochondria, caused perturbation in mitophagy and increased cell death (Guo et al., 2016). Furthermore, disorders of the mitochondrial dynamics lead to failure of mitophagy. A toxic effect of mHTT could be to compromise ubiquitin–proteasome activity (Wanker et al., 2019), but mHTT aggregates impair also transport of mitochondria in axons (Chang et al., 2006). To this end, recent studies identify HTT and adaptor protein huntingtin-associated protein-1 (HAP-1) as regulators of autophagosome transport in neurons, hypothesizing that an abnormal stabilization of the mHTT–HAP-1 interaction through the expanded polyQ tract may disrupt the movement of autophagosomes to cell bodies and lead to inefficient clearance of mitochondrial fragments in neurons (Wong and Holzbaur, 2014). Thus, the modulation of molecular pathways that include mitochondrial dysfunction, oxidative stress, and process of autophagy might represent very valuable therapeutic targets. Additionally, there is a need for reliable biomarkers to assess disease progression and to evaluate therapeutic interventions, especially in view of the upcoming HTT-lowering strategies, and mitochondrial signatures in HD could be used as potential biomarkers.

## Conclusion

*In vitro* and *in vivo* researches as well as studies in genetic models of PD revealed that mitochondrial dysfunction is not restricted to an imbalance in respiratory chain for ATP production with ROS generation. Failure of several mechanisms involved in mitochondrial health such as QC pathways, calcium homeostasis, and mitochondrial trafficking could cause cell death and neurodegeneration in PD and related disorders. Taking into account this idea, an important issue is whether a failure in mitochondrial homeostatic mechanisms is necessary and sufficient or only necessary but not sufficient to cause PD. Many PD genes are linked to mitochondrial dysfunction with a large number of them directly or indirectly involved in PINK1/Parkin pathway (Ryan et al., 2015). Many other PD genes are related to lysosomal system impairment. It is conceivable that the same genes involved in hereditary PD could exist, in the sporadic form of the disease, variants with weaker effects that could increase susceptibility to other external or internal factors thus leading to sporadic PD. Many of these genes likely act through PINK1/Parkin signaling (Truban et al., 2017).

Another question is about the primary event in mitochondrial dysfunction. Experimental models with cell cultures allow to study exclusively each single mitochondrial pathway involved in the homeostasis of the organelle. Nevertheless, it is conceivable that these multiple processes described are strictly interconnected and interact almost simultaneously, making difficult to discriminate the initiating event. Considering the high phenotypic variability of PD, different forms of disease (or the disease in different patients) could present different primary events in mitochondrial dysfunction, each converging anyway on a final way of action, which causes neuronal death. Nonetheless, in our opinion, the most important issue is whether mitochondrial dysfunction could represent the initiating factor in neurodegeneration. The finding of common mitochondrial dysfunctions, such as altered quality control mechanisms, imbalance in calcium homeostasis, impairment in trafficking in PD as well as in atypical parkinsonism and HD, the involvement of PINK1/Parkin both in PD and HD, and the possibility of producing animal models of all these neurodegenerative diseases by using toxins acting on mitochondrial respiratory chain, suggests that these events are probably important but not the trigger of neurodegeneration. We think that these events could feed neurodegeneration generating vicious cycles but could not represent the primary event. Instead, the observation that mitochondria could participate in degrading the proteins whose accumulation in cytosol generates pathological aggregates deserve great attention. The failure of this role and the impairment in import physiological proteins necessary for normal mitochondria functioning represent the strongest link of mitochondria with neurodegeneration. Nonetheless, mitochondrial impairment has a tight relationship with UPS and lysosome system. These factors are clearly demonstrated to be able to induce and/or worsen mitochondrial dysfunction in a vicious cycle.

Finally, the recognition in mitochondria of multiple pathways that could be affected in PD should lead to a different approach in therapeutic options for these diseases. Reflecting on the failure of drugs aiming to improve respiratory chain’s efficiency and scavenge ROS such as Co-Q10 and vitamin E (Weber and Ernst, 2006), a complex therapeutic approach considering the multiple processes that could cause mitochondrial impairment could be required.

## Author Contributions

VN substantially contributed to the conception and design of the manuscript and interpreting the relevant literature and drafted and revised the manuscript. GP and EP substantially contributed to the design of the manuscript and interpreting the relevant literature and drafted and revised the manuscript. MM revised the manuscript critically for important intellectual content. RC contributed to the design of the manuscript and revised it critically for important intellectual content. All authors contributed to the article and approved the submitted version.

## Conflict of Interest

The authors declare that the research was conducted in the absence of any commercial or financial relationships that could be construed as a potential conflict of interest.
